# Suicidal behaviors and sedentary lifestyles among adolescents: A cross-sectional epidemiological study in Latin American and Caribbean countries

**DOI:** 10.6061/clinics/2020/e2015

**Published:** 2020-11-02

**Authors:** Andressa Ferreira da Silva, Carlos Alencar Souza Alves Júnior, Patrícia de Fragas Hinnig, Luiz Rodrigo Augustemak de Lima, Diego Augusto Santos Silva

**Affiliations:** I Centro de Pesquisa de Cineantropometria e Desempenho Humano, Universidade Federal de Santa Catarina, FL, BR; II Laboratorio de Comportamento Alimentar, Universidade Federal de Santa Catarina, FL, BR; III Instituto de Educacao Fisica e Esportes, Universidade Federal de Alagoas, AL, BR

**Keywords:** Suicides, Mortality, Death, Sedentary Lifestyle, Adolescent Health, Mental Health

## Abstract

**OBJECTIVES:**

To verify the association between suicidal behaviors (ideation, planning, and attempts) and sedentary behaviors among adolescents from four Latin American and Caribbean countries.

**METHODS:**

A cross-sectional epidemiological study was conducted in four countries in Latin America and the Caribbean (Bahamas, Curação, El Salvador, and Guatemala). The sample comprised 6,813 adolescents aged 11-18 years, of which, 3,559 were females. The three suicidal behaviors considered were ideation, planning, and attempts. Sedentary behavior was regarded as the time that adolescents spent sitting, excluding time at school. Crude and adjusted logistic regression were used to estimate the odds ratio (OR) and 95% confidence interval (CI).

**RESULTS:**

Suicidal ideation was present in 10.7% of males and 22.7% of females. Suicidal planning was present in 8.6% of males and 16.3% of females. Suicidal attempt was present in 9.3% of males and 16.3% of females. Sedentary behavior was present in 39.6% of males and 45.7% of females. It was identified that male adolescents who reported spending ≥3 hours/day in sedentary behavior were more likely to have suicidal ideation (OR: 1.42; 95% CI: 1.13-1.80), whereas female adolescents who reported spending ≥3 hours/day in sedentary behavior were more likely to have suicidal ideation (OR: 1.55; 95% CI: 1.30-1.83), planning (OR: 1.54; 95% CI: 1.28-1.86), and attempts (OR: 1.31; 95% CI: 1.09-1.57).

**CONCLUSION:**

Adolescents of both sexes who reported spending ≥3 hours/day in sedentary behaviors were more likely to have some suicidal behaviors than those who spent less time in sedentary behaviors.

## INTRODUCTION

Suicide has been defined as a fatal self-injurious act with some evidence of intention to die (1). However, prior to performing this fatal act, the individual presents signs termed as suicidal behaviors, which necessarily develop from suicidal ideation, planning, and attempts (1). Suicide which is considered a public health problem, is the cause of approximately 800,000 people dying each year worldwide, representing one suicide every 40 seconds, of which, 79% occur in low and middle-income countries (2). Suicide rates have increased globally over the past 45 years, and reached the figure of 62,000 adolescents (10-19 years) in 2016 (2). In the Americas, the deaths of 220,000 young people (10-24 years), are attributed to suicide each year, of which 65,000 occur in Latin America (2,3).

Adolescents’ mental health can be determined by their lifestyle habits (4). Systematic reviews have shown that unhealthy behaviors (marijuana, tobacco, and alcohol use); inappropriate eating habits (low fruit and vegetable intake, and fast food consumption); insufficient physical activity; and sedentary behaviors (≥3 hours) are risk factors for development of suicidal behaviors in adolescents (10-19 years) (5,6). Sedentary behavior is associated with the risk of depressive symptoms, psychological distress, and low self-esteem, regardless of physical activity levels (5,8). Moreover, sedentary individuals are more likely to have lower brain-derived neurotrophic factor (BDNF) concentration—a biological marker for suicidal behaviors—which may influence neural homeostasis, increased anxiety and aggression levels, and self-injurious actions (9,10).

Previous studies that analyzed the relationship between suicidal behaviors and sedentary lifestyle investigated the relationship of only one of the suicidal behaviors with sedentary lifestyle, i.e., either ideation (4,11-12) or attempts (8). Two studies analyzed the relationship between suicidal ideation and attempts with adolescents’ sedentary behavior (13-14), but reported the three behaviors grouped under the same score (15,16). However, this analytical approach was not conducive to the understanding of each of the stages of suicidal behavior that usually occur in the form of a process, in which, suicide is first ideated, followed by the formulation of a specific method of execution, and ending in a potentially self-injurious act with the intention of ending one's own life (17,18).

Thus, the present study’s aim was to verify the association between suicidal behaviors (ideation, planning, and attempts) and sedentary behaviors in adolescents from four Latin American and Caribbean countries. This information allowed identifying correlates of suicidal behaviors that are changeable in society.

## MATERIAL AND METHODS

### Study Design

This descriptive cross-sectional study forms part of the macro project developed at the Federal University of Santa Catarina titled “Lifestyle of adolescents in Latin America and the Caribbean: focus on physical activity determinants.”

### Context

For the development of this study, secondary data from the World Health Organization (WHO) (19) and Centers for Disease Control and Prevention (20) were analyzed from four Latin American and Caribbean countries: Bahamas, Curação, El Salvador, and Guatemala, which were chosen since they had the most recent information on adolescent lifestyle indicators when this study was planned (January 2019) (19). In addition, only these four countries had finalized data based on the modifications to the questionnaires applied in the Latin American and Caribbean countries from 2013 onwards and made the data available (19).

Bahamas—an island country and a member state of the Caribbean community—had 401,060 high-income inhabitants with a human development index (HDI) of 0.807 and life expectancy of 76 years in 2017 (21). Curação—an island country of the Lesser Antilles in the southern Caribbean Sea and the Dutch Caribbean region—had 160,175 inhabitants and was considered a high-income economy (using the proxy means test in the absence of HDI information), had a life expectancy of 78 years in 2017 (21). El Salvador—a Central American country—with 6,377,853 inhabitants was considered to have a low-middle income, with its HDI of 0.674 and life expectancy of 74 years in 2017 (21). Guatemala—a Central American country—with 16,913,503 inhabitants was considered to have a low-middle income, with its HDI of 0.650 and life expectancy of 74 years in 2017 (21).

### Population and Sample

The study population comprised students aged 11-18 years from each of these countries. Each country carried out a sampling process which considered two stages. In the first stage schools were selected, with a probability proportional to the size (number of students in the target age group). In the second stage, the classes were selected, so that the students in each selected class could be invited to participate in the research. The sampling process for all the surveyed countries followed the same sample selection pattern (19,20).

Sample size calculation was performed to estimate unknown prevalence (50%), with a 95% confidence interval (CI), an approximate sampling error of three percentage points, and a design effect of 2.0. In general, a sample of 1,770 students was estimated in each country. However, each country could estimate sampling taking into account the regions, districts, and/or states within their country, and calculate the sample considering these aspects, provided that, the minimum number (n=1,770) was respected. For this reason, depending on each country, the planned sample was different. Further details on sampling in each country considering regions, districts, and/or states can be found in the WHO publications (19). Given these parameters, a sample of 12,610 adolescents was estimated [Bahamas (n=1,770); Curação (n=3,330); El Salvador (n=2,176); and Guatemala (n=5,334)]. Due to the response rates in the respective countries [Bahamas (78%); Curação (83%); El Salvador (88%); Guatemala (82%)], the total sample was 10,411 students [Bahamas (n=1,357); Curação (n=2,765); El Salvador (n=1,915); and Guatemala (n=4,374)].

The inclusion criteria were: adolescents aged 11-18 years. Adolescents who refused to participate in the study and those who did not return the informed consent form signed by their parents/guardians were considered as refusals. Only adolescents who answered all the variables investigated in this study were considered in the analysis.

### Data collection

In all the countries, students completed an answer sheet that corresponded to the applied questionnaire. The answer sheet was directly read by the computer, which reduced the chance of tabulation errors (19,20). Data collection in each country took place under the coordination of the Ministry of Health and/or the Ministry of Education, so that these agencies selected data collection teams and conducted standardized training of the instrument based on WHO’s workshops and recommendations (19). Data collection in the Bahamas and El Salvador took place in 2013, whereas in Curação and Guatemala it took place in 2015. Data collection took approximately two months (19-20).

### Outcome variables

Each of the three suicidal behaviors considered: ideation, planning, and attempts, were self-reported through the following three questions selected from the Global School-based Student Health Survey (GSHS) questionnaire (19,20): 1) Suicidal ideation: “During the past 12 months, have you seriously considered attempting suicide?” 2) Suicidal planning: “During the past 12 months, have you made a plan about how you would attempt suicide?” and 3) Suicidal attempts: “During the past 12 months, how many times have you actually attempted suicide?” While the answer options for 1) and 2) were “yes” or “no,” the options for 3) were: “never,” “once,” “2-3 times,” “4-5 times,” or “≥6 times.” The results were categorized as: “no” (no suicide attempt) when the adolescents chose the option “never,” or “yes” (presence of suicidal attempts) when the other options were selected.

According to the penal codes of each of the countries analyzed in the present study, attempting suicide was considered a crime in the Bahamas where the person was subject to imprisonment, whereas in the other countries, suicide attempts were not considered a crime (22).

### Exposure variable

Sedentary behavior was assessed using a single question from the GSHS questionnaire (19-20): “How much time do you spend during a typical/usual day sitting and watching television, playing computer games, talking to friends, or in other activities such as reading or chatting?” The answer options were: “<1 hour/day,” “1-2 hours/day,” “3-4 hours/day,” “5-6 hours/day,” “7-8 hours/ day,” or “>8 hours/day.” In this question, the adolescents were instructed not to consider time spent sitting at school or doing their school homework at home. The results were categorized according to the number of hours in the sitting position: Adolescents with <3 hours were considered as not exhibiting sedentary risk behaviors (grouped response: “<1 hour/day” and “1-2 hours/day”), or other grouped response options: ≥3 hours corresponding to sedentary risk behaviors). This cutoff was chosen because other studies had previously used it, which would facilitate comparisons (8,12-15,23).

### Covariates

The covariates were age, food insecurity, eating habits, physical activity, bullying, presence of friends, and body mass index (BMI). These variables were chosen because they had been considered as confounding variables in previous studies on both sedentary and suicidal behaviors (8,23).

Age was continuously collected. Food insecurity was used as a proxy for income, as in a previous GSHS study (8), through the question: “In the last 30 days, how often have you starved because there was not enough food in your home?&quot; The answer options were classified as: “never,” “rarely or sometimes,” and “most of the time and always.”

Eating habits were assessed through three questions. The first question was on the intake of fast foods, to which, the answers were categorized as “no consumption” and “consumption.” The second and third questions were on the consumption of fruits and vegetables, respectively, and the answers to both these questions were summed to generate a variable that corresponded to the adequacy of fruit and vegetable intake considering “adequate” (5 or more servings per day of fruits and vegetables) and “inadequate” (other options), according to nutritional recommendations (24).

Physical activity was investigated through the question, “During the past 7 days, on how many days did you engage in physical activity for a total of at least 60 minutes/day?” The answer options were categorized as: “physically active” (7 days/week) and “insufficiently physically active” (< 7 days/week), according to the guidelines of physical activity for adolescents (25).

Variable presence of friends was assessed by asking, “How many friends do you have?” The answer options were: “0,” “1,” “2,” or “3 or more.” Answers were categorized as “no” (“0”) and “yes” (other options). Student bullying was investigated by asking, “During the past 30 days, how many days have you been bullied?” The response options were: “No day,” “<1 or 2 days,” “3-5 days,” “6-9 days,” “10-19 days,” “20-29 days,” or “30 days.” The answers were categorized as “no” (No day) and “yes” (other options).

BMI was calculated as [BMI=body mass (kg)/height^2^ (cm)] using the self-reported questions about body mass and height. BMI Z-scores were calculated and the adolescents were classified using cutoff points of ≥+1 and <+1 standard deviations as “overweight/obesity,” and “eutrophic,” respectively (26).

### Statistical analysis

Initially, descriptive analysis was performed through frequency distribution. Binary, crude, and adjusted logistic regression analysis were used to assess associations between outcomes (suicidal behaviors) and exposure to sedentary behavior among adolescents, estimating odds ratio (OR) and 95% CI. Each outcome was separately analyzed. In the adjusted model, all covariates were inserted. For each country, sample weight was calculated and considered in the analysis. Analyses were stratified by sex (male and female) and performed using the Statistical Package for Social Sciences software (IBM SPSS Statistics, Chicago, USA), version 22.0, at 5% significance level.

### Ethical aspects

In all the participating countries, the local Ethics Committee approved the research procedures. In addition, the schools authorized the research and all the participants agreed to participate in the survey. Furthermore, the students’ parents/guardians were informed, and their signatures were obtained on the Consent Forms (19,20).

## RESULTS

Of the 10,411 students whose data were collected in the macro project, the 6,813 who fulfilled the necessary variables for the research participated in this study. The sample comprised: 3,254 (47.8%) males—45.8% were from Guatemala, 27.2% were 14 years-old, and 63.4% had no food insecurity—and 3,559 (52.2%) females—44.5% were from Guatemala, 28.0% were 14 years-old, and 64.7% had no food insecurity. Further information on both sexes is shown in Table 1.

**Figure 1:**
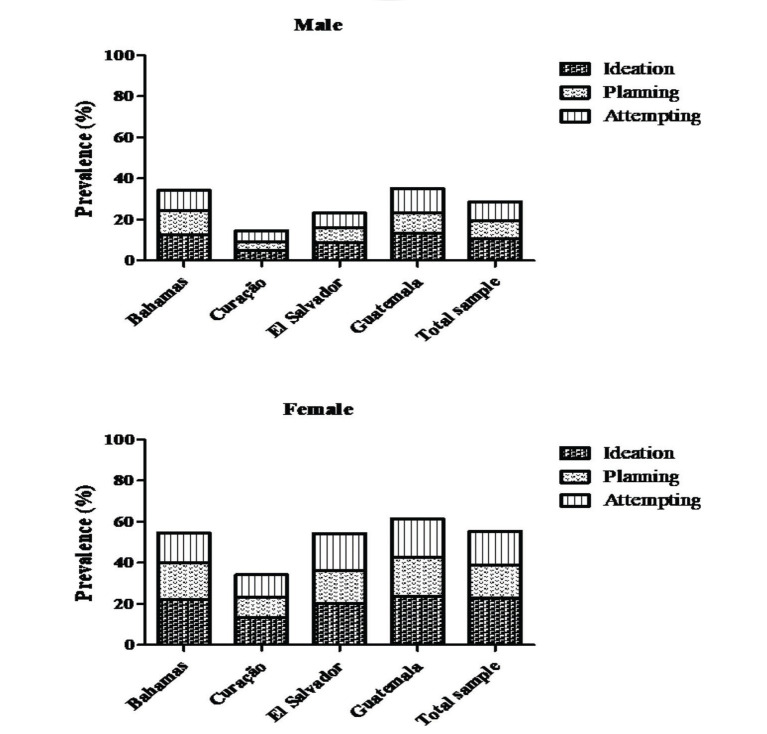
Sample distribution according to suicidal behaviors (ideation, planning, and attempts).

In male adolescents, suicidal ideation was present in 10.7% of the sample (12.5% Bahamas, 4.9% Curação, 8.6% El Salvador, and 13.4% Guatemala), while suicidal planning was present in 8.6% (11.8% Bahamas, 4.1% Curação, 7.5% El Salvador, and 10.0% Guatemala), and suicidal attempts in 9.3% (9.0% Bahamas, 5.6% Curação, 7.1% El Salvador, and 11.7% Guatemala) during the past 12 months. Among females, suicidal ideation was present in 22.7% of the sample (22.0% Bahamas, 13.5% Curação, 20.3% El Salvador, and 23.7% Guatemala), suicidal planning in 16.3% (18.0% Bahamas, 9.6% Curação, 16.1% El Salvador, and 19.0% Guatemala), and suicidal attempts in 16.3% (14.5% Bahamas, 11.0% Curação, 17.9% El Salvador, and 18.7% Guatemala) during the past 12 months (Figure 1).

**Figure 2:**
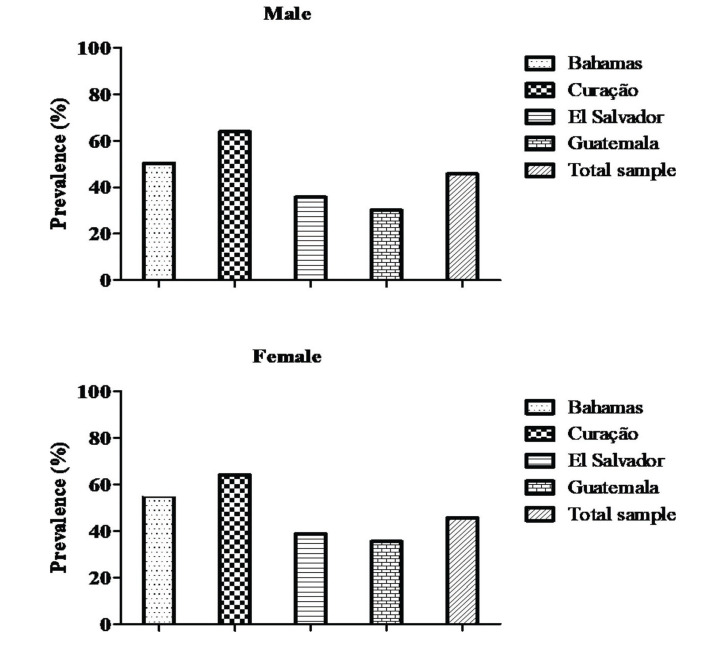
Sample distribution according to sedentary behavior.

Sedentary behavior was present in 39.6% of male adolescents (50.4% Bahamas, 63.9% Curação, 35.7% El Salvador, and 30.1% Guatemala) and 45.7% of female adolescents (59.6% Bahamas, 64.1% Curação, 38.8% El Salvador, and 35.5% Guatemala) (Figure 2).

It was identified in both the crude analysis (OR: 1.44; 95% CI: 1.15-1.80) and adjusted analysis (OR: 1.42; 95% CI: 1.13-1.80) that male adolescents who reported ≥3 hours/day in sedentary behavior were more likely to have suicidal ideation as compared to male adolescents, who spent a shorter time in sedentary behavior. Similarly when stratified by country, both the crude and adjusted analyses, showed that only male adolescents from the Bahamas and Guatemala who reported ≥3 hours/day in sedentary behavior were more likely to have suicidal ideation compared to male adolescents who spent a shorter time in sedentary behavior (Table 2).

In the crude analysis, female adolescents who reported ≥3 hours/day in sedentary behavior were more likely to have suicidal ideation (OR: 1.59; 95% CI: 1.35-1.87), planning (OR: 1.60; 95% CI: 1.34-1.91), and attempts (OR: 1.32; 95% CI: 1.11-1.58), when compared to adolescents who spent less than 3 hours/day in sedentary behavior. In the adjusted analysis, adolescents who reported ≥3 hours/day in sedentary behavior were more likely to have suicidal ideation (OR: 1.55; 95% CI: 1.30-1.83), planning (OR: 1.54; 95% CI: 1.28-1.86), and attempts (OR: 1.31; 95% CI: 1.09-1.57), when compared to adolescents who spent <3 hours/day in sedentary behavior. Both in the crude and adjusted analysis stratified by country, it was identified that female adolescents from the Bahamas, El Salvador, and Guatemala, who reported spending ≥3 hours/day in sedentary behavior were more likely to have suicidal ideation, planning, and attempts as compared to female adolescents who spent a shorter time in sedentary behaviors (Table 3).

## DISCUSSION

A study suggests that one third of adolescents with suicidal ideation will attempt suicide within a year (18). Thus, suicidal behaviors are considered risk factors for suicide. In the present study, among female adolescents suicidal ideation was present in 22.7% of the sample, while suicidal planning and attempts were each present in 16.3%, during the past 12 months. In studies conducted in the United States and Southeast Asian countries, female suicidal ideation ranged from 15.1%-23.4% (12-13,23), suicidal planning was 19.4%, and suicidal attempts were between 9.1% and 11.6% during the past 12 months (13,23). In a study conducted in 18 sub-Saharan African countries the median estimate of 12-month prevalence of self-harm was 16.9% in adolescents and adults (aged 10-25 years) (27). Among males, the presence of suicidal ideation, planning, and attempts were 10.7%, 8.6%, and 9.3%, respectively, during the past 12 months. Previous studies reported that during the past 12 months, the prevalence of suicidal ideation ranged from 9.3%-12.2% (13,23), while it was 9.8% (23) and 5.5%, for suicidal planning and attempts, respectively, (23) among male adolescents in the United States. Of the countries analyzed, Guatemala had the highest prevalence of suicidal behaviors in both sexes during the past 12 months, thus, corroborating estimates that 79% of suicides occur in low and middle-income countries (2).

In the present study, when considering a typical or usual day, 39.6% and 45.7% of male and female adolescents, respectively, reported ≥3 hours/day of sedentary behavior. These results were higher than those reported in studies conducted in the United States and Southeast Asian countries, where the prevalence ranged from 3.6%-12.8% for females and from 8.5%-13.3% for males (12,23). The prevalence of sedentary behavior in the present study was also higher than that reported in a systematic review that analyzed 43 countries from all regions of the world (28). A systematic review showed that 57% of adolescents of both sexes indulged in sedentary behavior after school, 26% of which was intended for television viewing and screen-based activities, and 54% for non-screen-based sedentary behaviors—motorized transport, homework, meals, and conversations with friends—which resulted in some of these behaviors occurring simultaneously (29). Male and female adolescents from high-income countries (Curação and Bahamas) showed almost twice the sedentary behavior as compared to those from low and middle-income countries (El Salvador and Guatemala), as reported in a study that analyzed 43 countries and found that middle and high-income countries generally had a higher prevalence of sedentary behaviors than low and middle-income countries (28).

In the present study it was found that female adolescents who reported having spent ≥3 hours/day in sedentary behavior were more likely to have suicidal ideation, planning, and attempts when compared to those who spent <3 hours/day in sedentary behavior. Similar results were found in a study conducted in 52 low and middle-income countries in which adolescents were 41%, 40%, and 25% more likely of having suicidal ideation, planning, and attempts, respectively, during the past 12 months, when compared to adolescents who reported <3 hours/day of sedentary behavior (30). Corroborating the present study, a survey conducted in the United States found that adolescents who spent >3 hours/day playing video games, or used computers for playing games were more likely to have suicidal ideation and planning. Moreover, the likelihood of having suicidal ideation increased for adolescents who had also watched television during the past 12 months (23). Studies have also shown that ≥3 hours/day of sedentary behavior was associated with a higher risk of suicidal attempts, regardless of gender (14,28). This association may reflect behavioral manifestations of internal depressive symptoms (e.g., stress and depression) (4), and content accessed on the internet and social media could contribute to different experiences related to suicide risk (23,31).

The association that this study found between sedentary and suicidal behaviors in females was independent of confounding factors (age, food insecurity, fruit and vegetable intake, fast food consumption, physical activity, adolescent bullying, presence of friends, and body mass index). A previous study (5) had already summarized that factors such as participation in physical activity increase the risk of depressive symptoms, psychological distress, and low self-esteem (5). However, this meta-analysis suggests that reducing time spent in sedentary behavior and increasing the duration of physical activity may protect the mental health of adolescents by decreasing depression, and increasing happiness and life satisfaction (32). A recent study with adolescents from 52 low and middle-income countries reported that spending increased time in sedentary behavior was considered a higher risk behavior than being insufficiently active (30). In addition, media usage was found to be related to events such as bullying and suicide risk in adolescents of both sexes (23). Finally, there is a consensus that low energy expenditure (≤1.5 metabolic equivalents [METs]) over time in sedentary positions and behaviors can harm adolescents’ physical and mental health (7).

This study’s results relating to males were similar to a survey conducted in 2015 in the United States, in which, spending >3 hours/day playing video games or using the computer for playing games was associated with a higher likelihood of suicidal ideation during the past 12 months (23). However, the present study found no association between sedentary risk behavior, and suicide planning and attempts for males. A study by Rostad et al. (23) conducted in the United States identified a higher likelihood of suicidal planning among male adolescents who reported using video games or computers for ≥3 hours, and a lower likelihood of suicidal attempts among male adolescents who reported watching television for ≥3 hours/day, which indicates different directions of the association of sedentary behavior with suicidal behaviors. In a recent study conducted by Uddin et al. (30) with adolescents from 52 low and middle-income countries, spending ≥3 hours/day in sedentary behaviors was associated with a 45% likelihood of presenting suicidal ideation and a likelihood of 29% each in planning and attempting suicide when compared to adolescents who reported <3 hours/day of sedentary behavior during the past 12 months. Males’ ways of dealing with suicidal behaviors are distinct from that of females. While females generally contemplate about the different stages of suicidal behavior; males may act impulsively in times of crisis, triggered by difficulties in coping with a momentary stressful situation without prior suicidal behaviors (18). This may explain why the present study found no association between suicidal planning and attempts with sedentary behavior in males.

When the analyses were stratified by country, it was found that female adolescents from Guatemala, the Bahamas, and El Salvador who spent ≥3 hours/day in sedentary behavior had greater odds of suicidal ideation and planning than those who spent less time in sedentary behavior. In relation to suicide attempts, they were more common among females from the Bahamas and El Salvador. For males, an association was observed between longer sedentary behavior (≥3 hours/day) and suicidal ideation in Guatemala and the Bahamas. To understand these differences between countries, it is necessary to discern the psychopathological, cultural, religious, and philosophical dimensions of self-injurious acts, which may differ according to each country (33). For example, 79% of global suicides occur in low and middle-income countries (2), as is the case in Guatemala and El Salvador. The present study also found associations between sedentary behavior (≥3 hours/day) and some suicidal behaviors in the Bahamas, considered a high-income country which considers suicide a crime and whose penal code prescribes imprisonment for people who attempt suicide (23). This indicates that suicidal behavior is not prevented, even if it is considered a crime in a certain country (22). Suicidal behaviors are a consequence of the social and economic context and to adjudge it as a crime is to prevent public health actions and measures to raise social awareness about certain behaviors (22,23). In this sense, suicidal behaviors are a public health problem and need to be perceived as such, so that individuals can prevent and/or treat these behaviors appropriately. The present study adds information to the literature on sedentary behavior—a factor related to suicidal behaviors—that can be avoided with health promotion actions, regardless of the income of different countries.

This study’s strengths include the analysis of different suicidal behaviors (ideation, planning, and attempts) in a few studies (30,31). Another strength is its sample size and scope, which represents four countries in Latin America and the Caribbean. However, it has limitations such as its cross-sectional design that does not allow establishing a cause and effect relationship, its estimation of sedentary behavior using subjective measures (questionnaire) that may have been affected by memory bias, and the impossibility of testing the cutoff point of ≤2 hours for sedentary behavior because the questionnaire had no intermediate alternative. Another limitation was that it did not analyze sedentary behavior activities separately.

It was concluded through an analysis of the last 12 months that adolescents of both sexes who reported ≥3 hours/day of sedentary behavior were more likely to have suicidal ideation compared to those who spent less time in sedentary behavior. Suicidal behaviors, such as planning and attempts were associated with ≥3 hours of sedentary behavior in female adolescents, regardless of confounding factors.

## AUTHOR CONTRIBUTIONS

da Silva AF and Júnior CA participated in the study’s conception, design, analysis, and writing of the manuscript. Hinning PF and de Lima LR were responsible for providing design and technical analysis support. Silva DA was responsible for the study’s conception, data curation, project administration, and writing of the manuscript. All authors contributed to revising the different versions of the manuscript for important intellectual content based on their expertise and background, and approved the final manuscript.

## Figures and Tables

**Table 1:** Descriptive characteristics of the sample.

	Male					Female				
	Bahamas	Curação	El Salvador	Guatemala	Total sample	Bahamas	Curação	El Salvador	Guatemala	Total sample
	n (%)	n (%)	n (%)	n (%)	n (%)	n (%)	n (%)	n (%)	n (%)	n (%)
Age (years)										
11	02 (0.5)	00 (0.0)	03 (0.4)	03 (0.2)	08 (0.2)	04 (0.8)	03 (0.4)	02 (0.3)	02 (0.1)	11 (0.3)
12	77 (18.6)	41 (7.7)	11 (1.3)	57 (3.8)	186 (5.7)	93 (17.5)	58 (7.8)	23 (3.3)	64 (4.0)	238 (6.7)
13	163 (39.3)	63 (11.8)	129 (15.8)	305 (20.5)	660 (20.3)	203 (38.2)	79 (10.7)	145 (20.6)	352 (22.2)	779 (21.9)
14	117 (28.2)	80 (15.0)	298 (36.6)	390 (26.2)	885 (27.2)	160 (30.1)	95 (12.9)	248 (35.3)	493 (31.1)	996 (28.0)
15	44 (10.6)	95 (17.8)	247 (30.3)	454 (30.5)	840 (25.8)	64 (12.0)	122 (16.5)	197 (28.0)	454 (28.6)	837 (23.5)
16	12 (2.9)	66 (12.4)	127 (15.6)	203 (13.6)	408 (12.5)	08 (1.5)	94 (12.7)	88 (12.5)	153 (9.7)	343 (9.6)
17	00 (0.0)	80 (15.0)	00 (0.0)	56 (3.8)	136 (4.2)	00 (0.0)	106 (14.3)	00 (0.0)	49 (3.1)	155 (4.4)
18	00 (0.0)	109 (20.4)	00 (0.0)	22 (1.5)	131 (4.0)	00 (0.0)	182 (24.6)	00 (0.0)	18 (1.1)	200 (5.6)
Food insecurity										
Never	213 (51.3)	412 (77.2)	532 (65.3)	905 (60.7)	2062 (63.4)	279 (52.4)	567 (76.7)	463 (65.9)	992 (62.6)	2301 (64.7)
Rarely or sometimes	179 (43.1)	108 (20.2)	267 (32.8)	562 (37.7)	1116 (34.3)	217 (40.8)	152 (20.6)	221 (31.4)	560 (35.3)	1150 (32.3)
Most of the times and always	23 (5.5)	14 (2.6)	16 (2.0)	23 (1.5)	76 (2.3)	36 (6.8)	20 (2.7)	19 (2.7)	33 (2.7)	108 (3.0)
Fruit and vegetable intake										
Adequate	69 (16.6)	76 (14.2)	163 (20.0)	368 (24.7)	676 (20.8)	70 (13.2)	87 (11.8)	141 (20.1)	455 (28.7)	753 (21.2)
Inadequate	346 (83.4)	458 (85.8)	652 (80.0)	1122 (75.3)	2578 (79.2)	462 (86.8)	652 (88.2)	562 (79.9)	1130 (71.3)	2806 (78.8)
Fast food consumption										
No consumption	128 (30.8)	179 (33.5)	370 (45.4)	576 (38.7)	1253 (38.5)	136 (25,6)	228 (30.9)	294 (41.8)	548 (34.6)	1206 (33.9)
Consumption	287 (69.2)	355 (66.5)	445 (54.6)	914 (61.3)	2001 (61.5)	396 (74.4)	511 (69.1)	409 (58.2)	1037 (65.4)	2353 (66.1)
Physical activity										
Physically active	80 (19.3)	80 (15.0)	145 (17.8)	275 (18.5)	580 (17.8)	59 (11.1)	66 (8.9)	68 (9.7)	175 (11.0)	368 (10.3)
Insufficiently physically active	335 (80.7)	454 (85.0)	670 (82.2)	1215 (81.5)	2674 (82.2)	473 (88.9)	673 (91.1)	635 (90.3)	1410 (89.0)	3191 (89.7)
Presence of friends										
Yes	381 (91.8)	499 (93.4)	771 (94.6)	1380 (92.6)	3031 (93.1)	495 (93.0)	689 (93.2)	683 (97.2)	1490 (94.0)	3357 (94.3)
No	34 (8.2)	35 (6.6)	44 (5.4)	110 (7.4)	223 (6.9)	37 (7.0)	50 (6.8)	20 (2.8)	95 (6.0)	202 (5.7)
Bullying										
No	331 (79.8)	417 (78.1)	641 (78.7)	1184 (79.5)	2573 (79.1)	421 (79.1)	585 (79.2)	529 (75.2)	1306 (82.4)	2841 (79.8)
Yes	84 (20.2)	117 (21.9)	174 (21.3)	306 (20.5)	681 (20.9)	111 (20.9)	154 (20.8)	174 (24.8)	279 (17.6)	718 (20.2)
Body mass index										
Eutrophic	223 (53.7)	319 (59.7)	555 (68.1)	998 (67.0)	2095 (64.4)	247 (46.4)	471 (63.7)	473 (67.3)	991 (62.5)	2182 (61.3)
Overweight/obesity	192 (46.3)	215 (40.3)	260 (31.9)	492 (33.0)	1159 (35.6)	285 (53.6)	268 (36.3)	230 (32.7)	594 (37.5)	1377 (38.7)
Total sample	415 (100.0)	534 (100.0)	815 (100.0)	1490 (100.0)	3254 (100.0)	532 (100.0)	739 (100.0)	703 (100.0)	1585 (100.0)	3559 (100.0)

**Table 2:** Logistic regression analysis between sedentarism and suicidal behaviors (ideation, planning, and attempts) in male adolescents from Latin American and Caribbean countries - Bahamas, Curação, El Salvador, and Guatemala.

		Crude analysis		Adjusted analysis*	
Variables	n (%)	OR (IC 95%)	*p*	OR (IC 95%)	*p*
**Ideation**					
Total sample					
Sedentarism			0.0015		0.0030
<3 hours	182 (52.4)	100		100	
≥3 hours	165 (47.6)	1.44 (1.15-1.80)		1.42 (1.13-1.80)	
Bahamas					
Sedentarism			0.0224		0.0097
<3 hours	18 (34.6)	100		100	
≥3 hours	34 (65.4)	2.03 (1.11-3.72)		2.37 (1.23-4.57)	
Curação					
Sedentarism			0.0743		0.0999
<3 hours	05 (19.2)	100		100	
≥3 hours	21 (80.8)	2.47 (0.91-6.65)		2.38 (0.85-6.70)	
El Salvador					
Sedentarism			0.1930		0.3677
<3 hours	40 (57.1)	100		100	
≥3 hours	30 (42.9)	1.39 (0.85-2.29)		1.27 (0.75-2.14)	
Guatemala					
Sedentarism			0.0009		0.004
<3 hours	119 (59.8)	100		100	
≥3 hours	80 (40.2)	1.68 (1.23-2.29)		1.61 (1.17-2.23)	
**Planning**					
Total sample					
Sedentarism			0.0629		0.1201
<3 hours	155 (55.2)	100		100	
≥3 hours	126 (44.8)	1.26 (0.99-1.62)		1.22 (0.95-1.58)	
Bahamas					
Sedentarism			0.3134		0.1765
<3 hours	21 (42.9)	100		100	
≥3 hours	28 (57.1)	1.36 (0.75-2.49)		1.54 (0.82-2.88)	
Curação					
Sedentarism			0.6351		0.7108
<3 hours	09 (40.9)	100		100	
≥3 hours	13 (59.1)	0.81 (0.34-1.93)		0.84 (0.34-2.09)	
El Salvador					
Sedentarism			0.0861		0.2071
<3 hours	33 (54.1)	100		100	
≥3 hours	28 (45.9)	1.58 (0.94-2.68)		1.42 (0.82-2.46)	
Guatemala					
Sedentarism			0.0234		0.1148
<3 hours	92 (61.7)	100		100	
≥3 hours	57 (38.3)	1.50 (1.06-2.13)		1.34 (0.93-1.93)	
**Attempts**					
Total sample					
Sedentarism					
<3 hours	170 (55.9)	100	0.0972	100	0.1529
≥3 hours	134 (44.1)	1.22 (0.96-1.55)		1.20 (0.93-1.53)	
Bahamas					
Sedentarism			0.2721		0.1405
<3 hours	17 (41.5)	100		100	
≥3 hours	24 (58.5)	1.44 (0.75-2.77)		1.69 (0.84-3.42)	
Curação					
Sedentarism			0.4004		0.7325
<3 hours	13 (43.3)	100		100	
≥3 hours	17 (56.7)	0.73 (0.34-1.53)		0.87 (0.40-1.90)	
El Salvador					
Sedentarism			0.2242		0.3954
<3 hours	33 (56.9)	100		100	
≥3 hours	25 (43.1)	1.40 (0.81-2.40)		1.28 (0.72-2.26)	
Guatemala					
Sedentarism			0.0078		0.0584
<3 hours	107 (61.1)	100		100	
≥3 hours	68 (38.9)	1.56 (1.12-2.16)		1.39 (0.99-1.96)	

OR: Odds ratio; 95% CI: 95% Confidence Interval; *Adjusted for covariates age, food insecurity, fruit and vegetable intake, fast food intake, physical activity, bullying, presence of friends, and body mass index.

**Table 3:** Logistic regression analysis between sedentarism and suicidal behaviors (ideation, planning, and attempts) in female adolescents from Latin American and Caribbean countries - Bahamas, Curação, El Salvador and Guatemala.

		Crude analysis		Adjusted analysis*	
Variables	n (%)	OR (IC 95%)	*p*	OR (IC 95%)	*p*
**Ideation**					
Total sample					
Sedentarism			<0.0001		<0.0001
<3 hours	332 (45.2)	100		100	
≥3 hours	403 (54.8)	1.59 (1.35-1.87)		1.55 (1.30-1.83)	
Bahamas					
Sedentarism			0.0006		0.0009
<3 hours	31 (26.5)	100		100	
≥3 hours	86 (73.5)	2.21 (1.40-3.48)		2.21 (1.38-3.55)	
Curação					
Sedentarism			0.2767		0.4247
<3 hours	31 (31.0)	100		100	
≥3 hours	69 (69.0)	1.29 (0.82-2.02)		1.22 (0.75-2.00)	
El Salvador					
Sedentarism			<0.0001		<0.0001
<3 hours	65 (45.5)	100		100	
≥3 hours	78 (54.5)	2.25 (1.55-3.26)		2.17 (1.47-3.21)	
Guatemala					
Sedentarism			<0.0001		<0.0001
<3 hours	205 (54.7)	100		100	
≥3 hours	170 (45.3)	1.73 (1.37-2.19)		1.64 (1.28-2.10)	
**Planning**					
Total sample					
Sedentarism					
<3 hours	259 (44.6)	100	<0.0001	100	<0.0001
≥3 hours	322 (55.4)	1.60 (1.34-1.91)		1.54 (1.28-1.86)	
Bahamas					
Sedentarism			0.0018		0.0026
<3 hours	25 (26.0)	100		100	
≥3 hours	71 (74.0)	2.19 (1.34-3.59)		2.19 (1.32-3.66)	
Curação					
Sedentarism	22 (31.0)		0.3687		0.6649
<3 hours	49 (69.0)	100		100	
≥3 hours		1.27 (0.75-2.16)		1.13 (0.65-1.96)	
El Salvador					
Sedentarism			0.0016		0.0048
<3 hours	54 (47.8)	100		100	
≥3 hours	59 (52.2)	1.92 (1.28-2.88)		1.84 (1.21-2.82)	
Guatemala					
Sedentarism			<0.0001		<0.0001
<3 hours	158 (52.5)	100		100	
≥3 hours	143 (47.5)	1.87 (1.45-2.41)		1.77 (1.36-2.31)	
**Attempts**					
Total sample					
Sedentarism					
<3 hours	281 (48.4)	100	0.0019	100	0.0045
≥3 hours	299 (51.6)	1.32 (1.11-1.58)		1.31 (1.09-1.57)	
Bahamas					
Sedentarism			0.0060		0.0050
<3 hours	20 (26.0)	100		100	
≥3 hours	57 (74.0)	2.14 (1.24-3.68)		2.25 (1.28-3.97)	
Curação					
Sedentarism			0.7973		0.9890
<3 hours	28 (34.6)	100		100	
≥3 hours	53 (65.4)	1.07 (0.66-1.73)		100 (0.60-1.65)	
El Salvador					
Sedentarism			0.0001		0.0004
<3 hours	58 (46.0)	100		100	
≥3 hours	68 (54.0)	2.13 (1.44-3.14)		2.07 (1.38-3.11)	
Guatemala					
Sedentarism			0.0309		0.0743
<3 hours	175 (59.1)	100		100	
≥3 hours	121 (40.9)	1.33 (1.03-1.72)		1.28 (0.98-1.67)	

OR: Odds ratio; 95% CI: 95% Confidence Interval; *Adjusted for covariates age, food insecurity, fruit and vegetable intake, fast food intake, physical activity, bullying, presence of friends, and body mass index.
